# Effective Therapeutic Approach for Head and Neck Cancer by an Engineered Minibody Targeting the EGFR Receptor

**DOI:** 10.1371/journal.pone.0113442

**Published:** 2014-12-01

**Authors:** Young Pil Kim, Dongsun Park, Jae Jin Kim, Won-Jae Choi, Sun Hee Lee, Seo Yun Lee, Soyeon Kim, Jee Min Chung, Jinseon Jeon, Byoung Dae Lee, Joo-Ho Shin, Yun-il Lee, Hyeseong Cho, Jeong-Min Lee, Ho Chul Kang

**Affiliations:** 1 Department of Bio-Engineering, Life Science RD Center, Sinil Pharmaceutical Co., Seongnam, South Korea; 2 Department of Physiology, Ajou University School of Medicine, Suwon, South Korea; 3 Department of Biomedical Sciences, The Graduate School, Ajou University, Suwon, South Korea; 4 Department of Neuroscience, Kyung Hee University School of Medicine, Seoul, South Korea; 5 Division of Pharmacology, Department of Molecular Cell Biology, Samsung Biomedical Research Institute, SungKyunKwan University School of Medicine, Suwon, South Korea; 6 Well Aging Research Center, Samsung Advanced Institute of Technology (SAIT), Suwon, South Korea; 7 Department of Biochemistry, Ajou University School of Medicine, Suwon, South Korea; Johns Hopkins University, United States of America

## Abstract

Cetuximab, a chimeric monoclonal antibody developed for targeting the Epidermal Growth Factor Receptor (EGFR), has been intensively used to treat cancer patients with metastatic colorectal cancer and head and neck cancer. Intact immunoglobulin G (IgG) antibody like cetuximab, however, has some limitations such as high production cost and low penetration rate from vasculature into solid tumor mass due to its large size. In attempt to overcome these limitations, we engineered cetuximab to create single chain variable fragments (scFv-CH3; Minibody) that were expressed in bacterial system. Among three engineered minibodies, we found that MI061 minibody, which is composed of the variable heavy (V_H_) and light (V_L_) region joined by an 18-residue peptide linker, displays higher solubility and better extraction properties from bacterial lysate. In addition, we validated that purified MI061 significantly interferes ligand binding to EGFR and blocks EGFR's phosphorylation. By using a protein microarray composed of 16,368 unique human proteins covering around 2,400 plasma membrane associated proteins such as receptors and channels, we also demonstrated that MI061 only recognizes the EGFR but not other proteins as compared with cetuximab. These results indicated that engineered MI061 retains both binding specificity and affinity of cetuximab for EGFR. Although it had relatively short half-life in serum, it was shown to be highly significant anti-tumor effect by inhibiting ERK pathway in A431 xenograft model. Taken together, our present study provides compelling evidence that engineered minibody is more effective and promising agent for *in vivo* targeting of solid tumors.

## Introduction

The epidermal growth factor receptor (EGFR) is one of ErbB family of receptor tyrosine kinases [1]. Ligand-mediated EGFR signaling such as either PI3K/AKT or RAS/ERK pathway regulates various cellular processes including cell survival, death, growth, proliferation, and motility [2]. Dysregulation of EGFR by its overexpression or mutation leads to development of a wide range of epithelial cancers, e.g. breast, colon, head and neck, kidney, lung, pancreas, and prostate cancer [3]–[5]. This is a rationale for the development of EGFR interferent as antitumor agents in the cancer therapy [5]. Last decade, two major classes of EGFR inhibitor have been developed to target the EGFR. The first class, tyrosine kinase inhibitors including gefitinib and erlotinib, acts by competitively binding to the ATP pocket of EGFR. The second class, monoclonal antibodies such as cetuximab and panitumumab, can interfere ligand binding of EGFR [6], [7]. Both classes of agents display significant anti-tumor activity in a range of EGFR-dependent mouse xenograft models [7], [8]. Especially, cetuximab, a monoclonal antibody targeting EGFR, has been intensively studied as an anti-cancer agent approved by the FDA for treating head and neck cancer [9].

The targeted therapy using intact IgGs like cetuximab has notably improved poor prognosis and overall survival in cancer patients [10]. However, in spite of their high antitumor efficacy, the use of intact whole IgGs for cancer therapy is limited due to high production costs, because of the requirement for a mammalian expression system, and poor penetration rate into tumor tissues [11]. Hence, the imperious need for engineered antibody produced from bacterial system has been increased and a bunch of studies have introduced various structures of engineered minibody base on the diversity of intact IgG [11], [12].

To overcome current issues, we generated single chain variable fragments (scFvs) expressed in *E.coli* based on parental antibody, cetuximab. The engineered scFvs has not only the domain order of V_H_ and V_L_ region but also flexible polypeptide linker composed of 18 amino acid residues between V_H_ and V_L_ domains for the effective production and stability. The scFv (hereafter minibody) region was connected with C_H_3 *via* hinge region. In the present study, the engineered minibody (V_H_-18Linker-V_L_-Hinge-C_H_3) was characterized *in vitro* and compared with cetuximab for its *in vivo* application in xenograft model.

## Materials and Methods

### Construction of MI045, MI053 and MI061 expression vectors

To construct the MI045 (VL-Linker-VH), the DNA fragments encoding VL domain and VH were synthesized based on the amino acid sequences (1st-107th aa) of the Chain A of Cetuximab Fab fragment (GenBank accession no. 1YY8_A) and the amino acid sequences (1st-119th aa) of the Chain B of Cetuximab Fab fragment (GenBank accession no. 1YY8_B), respectively. The classical (G4S)3 sequences (GGGGSGGGGSGGGGS) were used as a linker [13]. The MI053 was created by a similar method except that the length and sequences of the linker, consisted of 18 aa (GSTSGSGKPGSGEGSTKG) derived from m218 Whitlow linker [14]. The MI061 has same structure in comparison with MI053 except domain order of VH and VL. The DNA fragment encoding a Hinge-CH3 domain was also synthesized based on the amino acid sequences of human IgG-1. The Hinge sequence was modified to 15 aa (EPKSPKSADKTHTAP). The codons were optimized for *E. coli* expression. Each scFv DNA fragments were digested with *BamHI* and *HindIII* and the Hinge-C_H_3 DNA fragment digested with *HindIII* and *XhoI* were inserted into pET26b. These constructs contained pelB leader sequence at N-terminal end for periplasmic expression in BL21 (DE3), and contained 6-Histidine residues at C-terminal end for affinity purification.

### Expression and purification of minibody

Each construct coding for MI045, MI053 and MI061 was transformed into *E. coli* BL21 (DE3). Grown colonies were cultured at 37°C for overnight with vigorous agitation, and then 1 ml of this pre-culture was inoculated into fresh 1 L LB broth. Protein expression was induced by the addition of IPTG at a final concentration of 0.4 mM when the optical density at 600 nm reached 0.5–0.6. The cultures were harvested and resuspended with 100 ml of PBS buffer containing 1 mM PMSF, 0.2 mg/ml DNase I, and 0.2 mg/ml RNase A, and then disrupted by sonication. The soluble protein extracts were obtained by centrifugation at 20,000 rpm for 20 min at 4°C and incubated with Ni^2+^-NTA resin column (GE Healthcare Life Science, USA) according to the supplier's manual. The column was washed with 30 column volumes of PBS containing 50 mM Imidazole, and then bound proteins were eluted with 10 column volumes of PBS containing 400 mM Imidazole. The purified proteins were analyzed by 12% SDS-PAGE. The protein concentrations were determined by the bicinchoninic acid method (Pierce, Rockford, IL, USA).

### Human protein microarray

The HuProt Human Proteome Microarray was purchased from CDI laboratories (http://www.cdi-lab.com). The Chip includes 16,368 unique full-length human recombinant proteins in duplicate along with several control proteins such as IgG, GST, BSA-biotin, and histones as previously described [15]. Antigen binding specificity of both minibody and cetuximab (Erbitux, Merck, Darmstadt, Germany) were measured by using Alexa-Fluor 546 or 647 conjugated anti-human IgG antibody (Invitrogen, Carlsbad, CA). Mouse anti-GST monoclonal antibody (Invitrogen) was used as control. Briefly, the protein chip was first incubated with blocking buffer (5% BSA in PBS with 0.05% (v/v) Tween 20) for 30 min at RT and then minibody (32 nM), cetuximab (32 nM) or mouse anti-GST monoclonal antibody were further incubated under the lifterslip (Thermo scientific, USA) for 1 hr at RT. After washing three times with 1x PBS containing 0.05% Tween 20 by gentle shaking for 10 min each, the microarray was incubated with Alexa-Fluor conjugated antibody. Subsequently, the microarray was washed three times and then the values of probe signal were obtained using a GenePix Pro 6.0 software (Molecular Devices, Sunnyvale, CA).

### Cell culture and reagents

The A431 (KCLB, Seoul, Korea) cells were cultured in RPMI 1640 medium (Gibco, Karlsruhe, Germany) supplemented with 10% fetal bovine serum (Hyclone, Logan, UT, USA), 100 U/ml penicillin and 100 µg/ml streptomycin (Sigma, St. Louis, MO, USA). The cells were grown at 37°C, 5% CO_2_ in a humidified atmosphere.

### Flow cytometric analysis for antibody binding assay

To confirm cetuximab, MI053 and MI061 binding to EGFR, flow cytometry was performed using the A431 cell line that expresses high levels of EGFR. A total of 1×10^5^ cells were incubated with cetuximab, MI053 or MI061 at 2 ug/ml for 30 min on ice and washed twice with staining buffer (1% BSA/PBS), followed by a 30 min incubation with 4 ug/ml of a FITC-labeled goat anti human Fc mAb (Sigma). The cells were analyzed by an Accuri C6 flow cytometer (BD Biosciences, San Jose, CA, USA).

### The competitive binding assay

The A431 cells were adjusted to 1×10^5^ cells/tube and incubated containing 6 nM FITC-labeled EGF ligand (Molecular Probes, Leiden, Netherlands) plus the relevant cold competitors, which are cetuximab and MI061 (2 ug/ml) for 30 min at 4°C. Following washed three times to remove unbound materials, cells were analyzed by an Accuri C6 flow cytometer (BD Biosciences) to determine the relative amount of bound FITC-labeled EGF.

### Immunoassay

The 96 wells immunoplate (SPL, Seoul, Korea) were coated with sEGFR (Fitzgerlad Industries International, MA, USA) reconstituted in 200 mM Na_2_CO_3_ (pH 9.6). The plate was blocked with 200 ul/well of 1X Blocking solution (Sigma), sealed and stored 4°C until use. Dilutions of cetuximab (from 2 mg/ml) were prepared in a range of 1∶10-1∶4096000 by serial dilution with PBS for standard curve. For immunoassay of minibody activity, the diluted plasma (1∶50) was added into each well and the incubated at room temperature for 1 hr. Then, each well was washed three times with PBS-T (Phosphate Buffered Saline with 0.05% Tween 20) and 100 µl/well of 1∶100 mouse anti-human IgG (C_H_3 domain) antibody (Thermo Scientific, Rockford, IL, USA) was added. The each well was incubated for 1 hr and then anti-mouse IgG HRP (Sigma) secondary antibody was added. To evaluate the minibody concentration in plasma, the each well was washed three times with PBS-T and rabbit anti-human IgG Fab specific antibody (Sigma) was incubated for 1 hr along with anti-rabbit IgG HRP (Sigma) secondary. After washing with PBS-T, 100 µl/well TMB substrate (Sigma) was added to each well and incubated for 10 min at RT. The reaction was stopped by 1 M H_2_SO_4_ and plate was then read on a plate reader at 450 nm.

To determine the half-life of MI061, the blood samples were collected at 1, 3, 6, 24 and 48 hrs after the initial injection (i.p., 0.3 mg/mouse) and heparinized plasma samples were separated immediately by centrifugation (13,000 rpm for 5 min at 4°C). The concentration of minibody was estimated using non-compartmental methods with BA calc 2007 (KFDA, Seoul, Korea).

### Phosphorylation assay

Phosphorylation assays were performed on A431 cells in the presence of either cetuximab Fab (30 µg/ml) or minibody (30 µg/ml). After culturing for 8 hrs, cells were stimulated with 10 ng/ml EGF for 20 min at 37°C. Cells were lysed and the phosphorylated EGFR protein levels were measured using the PathScan Phospho-EGF Receptor (Tyr845) Sandwich ELISA Kit (Cell signaling) according to the manufacturer's instructions.

### Xenograft model

Six-to seven-week-old male athymic (nu/nu) mice were purchased from Nara biotech (Seoul, Korea). Mice were housed under pathogen-free conditions in microisolator cages with laboratory food and water available *ad libitum*. A431 xenografts were established by s.c. injecting between 5×10^6^ cells/100 µl PBS into the left flank of athymic mice. Tumor volume was calculated every 2-3 days with a digital caliper and using the following formula: Volume = length×width×height. Tumors were allowed to grow to a volume 100 mm^3^, and mice were randomized into groups of six animals each. Mice were treated with saline q3d, cetuximab at 0.25 mg/mouse (q3d), cetuximab Fab at 0.25 mg/mouse (q3d), and MI061 at 0.25 mg/mouse (q7d). All drugs were administrated by i.p. injection. Treatment of animals was continued for the duration of the study. At the end of each time point, the mice were euthanized with CO_2_ asphyxiation. Statistical analysis of tumor growth for each of the studies was determined by a *Student*'*s t test* using the computer program SigmaStat (Jandel, San Rafael, CA, USA). Mice were maintained in accordance with the policies of the Konkuk University Institutional Animal Care and Use Committee (IACUC). The study conducted herein was approved by the Konkuk University IACUC (Approval No.: KU14024).

### Immunohistochemisry (IHC)

Excised tumor tissues were preserved in 10% neutral buffered formalin for IHC staining. IHC staining procedures for total EGFR were as follows: Paraffin was depleted from a slide and incubated with pepsin solution for 15 min at 37°C. Tissue sections were covered with 3% H_2_O_2_ to block endogenous peroxidase for 10 min at room temperature. Slides were incubated with anti-EGFR antibody at 1∶100 (Cell Signaling Technology, IL, USA) for 18 hrs at 4°C. After washing with TBS, the slides were incubated with a secondary antibody (DAKO Japan, Kyoto, Japan) for 30 min at room temperature. Tissue staining was visualized using a DAB (3,3′diaminobenzidine) substrate chromogen solution. IHC staining for phosphorylated EGFR protein was conducted as follows: After the same pre-processing of the anti-EGFR antibody as described above, tissue sections were blocked with protein block serum-free to prevent nonspecific reactions for 10 min at room temperature. Slides were incubated with primary p-EGFR (Tyr845) antibody at 1∶50 (Cell signaling) for 18 hrs at 4°C. The slides were then incubated with secondary antibody (DAKO Japan) for 15 min at room temperature. Tissue staining was visualized using a DAB substrate chromogen solution. The slides were then counterstained with hematoxylin, dehydrated, and mounted.

### Western blot analysis

For western blot analysis of purified MI045 (0.23 µg/lane), MI53 (0.55 µg/lane) and MI061 (1.30 µg/lane), the protein samples were initially separated on 12% SDS-PAGE gels and transferred onto a nitrocellulose membrane (Thermo scientific, USA). The membrane was immunoblotted with primary anti-C_H_3 domain antibody A567H (Thermo Scientific, USA) and each protein was visualized by incubation with secondary HRP-conjugated anti-mouse IgG antibody. To check the expression levels of EGFR, p-EGFR, p-AKT, ERK and p-ERK in tumor cells by western blot analysis, tumor masses were removed from the flank of the mice and homogenized. The protein samples containing the same amount of total proteins (40 µg/well) were extracted and separated on 12% SDS-PAGE gels and then transferred onto a nitrocellulose membrane. The primary antibodies, EGF receptor (Cell signaling), phospho-EGF receptor (Tyr1068) (Cell signaling), AKT pS473 (Rockland), p44/42 MAPK (Erk1/2) (Cell signaling), and phospho-p44/42 MAPK (Erk1/2) (Thr202/Tyr204) antibody were used for immunoblotting according to the manufacturer's recommended ratio (1∶1000). The membrane was developed and visualized using Pierce ECL Plus Western Blotting Substrate (Thermo scientific, USA).

## Results

### Expression and characterization of engineered minibodies

**Figure 1:**
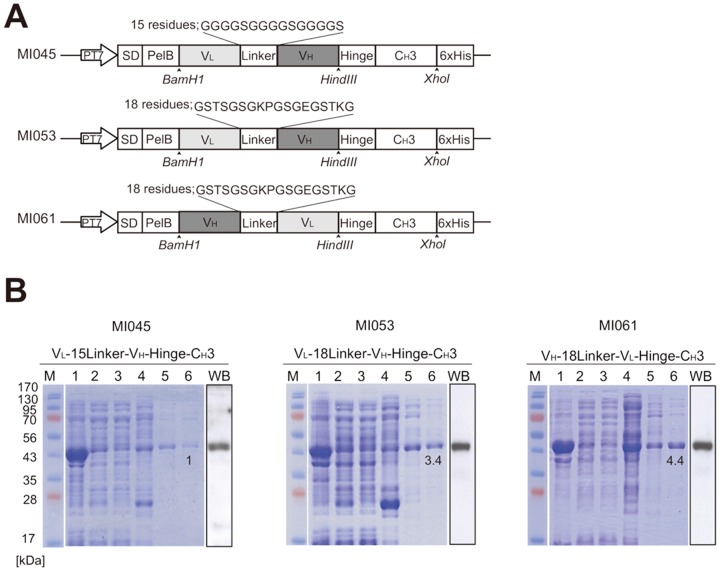
Purification of three engineered minibodies from bacterial system. (A) Schematic illustration of constructs coding each minibody. (B) Purification of His-tagged minibody from *E. coli* cells. BL21 (DE3) cells were transformed with each construct and minibody production was induced by addition of IPTG. His-tagged minibodies were purified using a Ni-NTA column. The eluted proteins were resolved on 12% SDS-PAGE and visualized by either Coomassie Brilliant Blue (CBB) or immunoblot assay with an anti-CH3 domain antibody. The numbers indicate recovery rate compared with MI045. M; molecular size marker, 1; crude extract, 2; soluble proteins, 3; pellet, 4; flow through, 5; washing with 50 mM imidazole, 6; elution with 400 mM imidazole, WB; western blot.

This minibody basically consists of the V_H_ and V_L_ domains joined by a flexible peptide linker, which can affect stability and solubility of antibody, and they were connected with C_H_3 *via* hinge region. Here, we generated three minibodies comprising different length of linker or reversely ordered V_H_ and V_L_ domains (Fig 1A). The first minibody that has relatively short linker was named MI045 and another minibody was named MI053 that they have 15 or 18 residues as a linker, respectively. Last one consists of swapped V_H_ and V_L_ domain, and named MI061. To explore the nature of three engineered minibodies, expression of each minibody was tested in *E. coli* as described by Hu et al. [16]. Soluble fraction of *E. coli* lysate applied to affinity chromatography and resulted in recovery of highly purified minibodies without any fragmentations in the western blot (Fig 1B). Each minibody showed the estimated molecular weight, and the recovery rate of MI053 and MI061 showed 3.4 or 4.4 folds higher than MI045, respectively. This data indicate that 18 residues of linker are indeed structurally adequate model than 15 residues to retain solubility. Interestingly, we found that the recovery efficiency of MI061 is greater than MI053 (Fig 1B). These data suggest that length of linker and domain order of variable chains, which in turn affect solubility of antibody, are associated with nature of minibody.

### Activity and specificity of engineered minibodies

**Figure 2:**
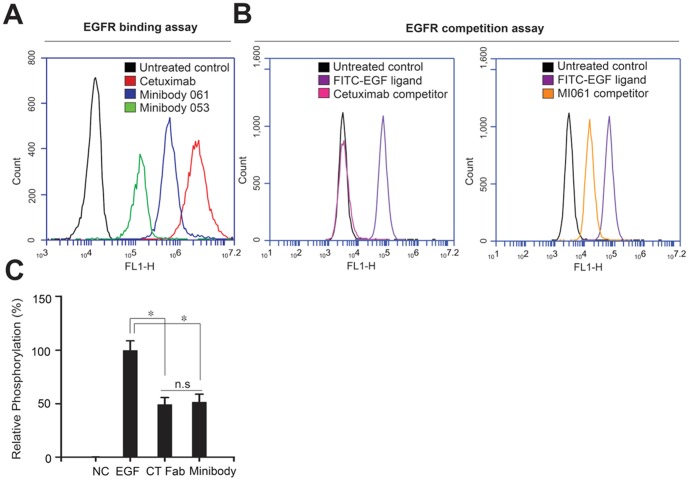
Antagonizing activity of minibody against EGFR. (A) EGFR binding assay. Each antibody was incubated with EGFR-overexpressed A431cells and then binding affinity to EGFR was analyzed by FL1-H intensity using flow cytometry (FACs) (B) EGFR competition assay. FITC-labeled EGF was incubated with A431 cells and its binding activity to EGFR was monitored by FACs. For the competition assay, EGF was incubated cells along with either cetuximab (left panel) or MI061 (right panel). (C) EGFR phosphorylation assay. The relative phosphorylation rate of EGFR induced by each antibody was evaluated by ELISA using an anti-EGFR phospho-antibody. The EGF was used as a control for the phosphorylation of EGFR as indicated. Quantified data are expressed as mean ± s. e. m. ^*^P<0.05. n.s, not significant.

**Figure 3:**
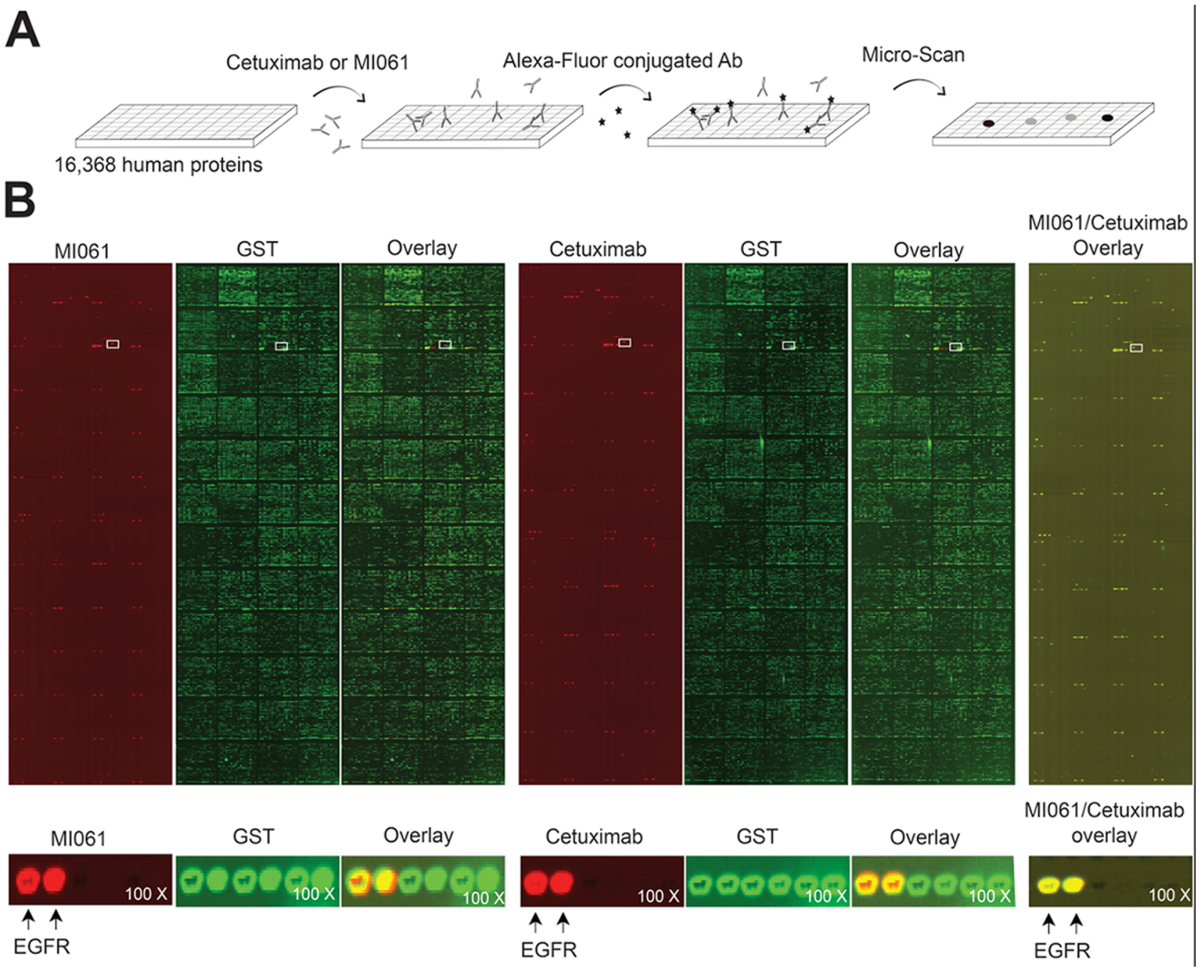
The EGFR binding specificity of MI061 minibody. (A) Schematic illustration of protein microarray process. (B) The protein chips were incubated with either MI061 or cetuximab, and antigen-antibody interactions were detected by using Alexa-Fluor conjugated secondary antibody. After washing, each chip was scanned by GenePix Scanner at 532 nm (Cy3) and 635 nm (Cy5). Each value of probe signals was obtained by using GenePix Pro 6.0 software. Red signal indicates antigen binding intensity of both MI061 and cetuximab. All GST-fusion proteins were detected by anti-GST antibody and visualized by green fluorescence as indicated. White rectangles indicate magnification area for bottom panel.

Based on the recovery yield of each minibody from bacterial lysate, both MI053 and MI061 were selected for the additional study. To measure their activity depending on domain orientation of variable regions, we initially assessed their binding activity to EGFR. A431 cells expressing high level of EGFR were incubated with antibodies, and the subsequent change in fluorescence signal was measured by a laser based flow cytometry analysis. Consequently, all treated antibodies elicited a shift in the fluorescence signal greater than that of the untreated control (Fig 2A). Among them, cetuximab showed the highest binding activity to EGFR. Particularly, we confirmed that MI061 and MI053 also retained high affinity and were capable of strong binding to EGFR. The affinity between MI061and EGFR was stronger than the affinity between MI053 and EGFR. These data indicated that domain order of variable chains is associated with affinity of minibody. An alternative approach to obtain the specificity, the competition assay was performed using a FITC-labeled EGF ligand with concept that interference of interaction between EGF and EGFR could inhibit EGFR activation and its downstream signaling pathway. As a result, cetuximab was entirely competed with EGF, whereas treatment of MI061 partially inhibited binding with EGFR (Fig 2B). To confirm inhibitory capability of MI061, we analyzed relative phosphorylation rate of EGFR (Fig 2C). Phosphorylation rate of EGFR induced by EGF was analyzed in A431 cells in presence of either cetuximab Fab fragment (CT Fab), EGFR binding regions of cetuximab, or MI061. The treatment of EGF with CT Fab suppressed relative phosphorylation to about 50%. Similarly, the treatment of EGF with MI061 also showed same effect in comparison with CT Fab. Although their binding affinity and specificity were relatively lower compared to cetuximab, MI061 was able to strongly recognize EGFR and suppress EGFR's phosphorylation in the cell system. To validate antigen binding specificity of MI061, we used the human protein microarray chip, which contains 16,368 unique GST-tagged full-length human proteins (Fig 3A). The protein chip was treated in parallel with either MI061 or cetuximab in equal molar ratio for 1 hr at RT. The proteins binding with antibody were detected by using Alexa-Fluor conjugated secondary antibody. The chip was then scanned using a GenePix 4100A scanner. To normalize the Alexa-Fluor signal, the protein chip was then probed with an anti-GST antibody, followed by scanning. In the each protein microarray chip, both MI061 and cetuximab showed similar binding pattern in duplicate with same intensity, respectively (Fig 3B). Also, in a high powered view, they only bound EGFR protein except for control proteins such as GST (Fig 3B, lower panel).

### Antitumor effect of engineered minibody

**Figure 4:**
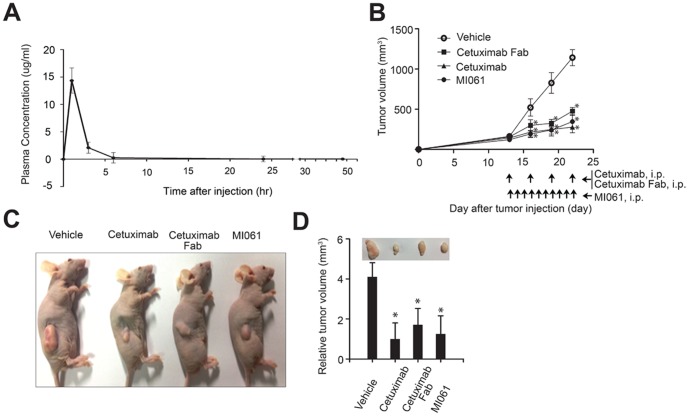
Antitumor effect of MI061 minibody. (A) Pharmacokinetics of MI061 minibody. Blood samples for the pharmacokinetics of MI061 were collected at 1, 3, 6, 24 and 48 hours after the initial intraperitoneal injection (i.p., 0.3 mg/mouse) and their concentration was estimated using non-compartment methods. (B) Time-course of the change in tumor size. Athymic nude mice were treated with vehicle or with cetuximab or with cetuximab Fab every 3 days, or with minibody daily for 10 days by i.p injection (0.25 mg/mouse). The treatment was started when the tumor size reached 100 mm^3^ after xenograft. The tumor size was measured with calipers during experiment days. (C) Representative findings of A431 tumor masses. (D). Relative tumor volume. The size of A431 tumor masses removed from athymic mice treated each antibody was measured with calipers and was indicated as relative volume compared with cetuximab treated group. Quantified data are expressed as mean ± s. e. m. ^*^P<0.05

To evaluate and characterize the plasma pharmacokinetics (PK) of MI061, PK parameters were examined in xenograft animal. After intraperitoneral (i.p.) injection of MI061 with 0.3 mg/inj, the C(max), T(max), area under the curve (AUC) and t_1/2_ were measured as 14.4 mg/L, 1 hr, 24.8 mg•h/L and 4.3 hr, respectively (Fig 4A). According to PK data of MI061, the antitumor effect of MI061 was monitored in the A431 xenografts model. Each of the mice was treated for 22 days from 13^th^ post-transplantation day through i.p. injection with cetuximab, CT Fab by every 3 days, or MI061 by daily base on the PK activity. Injection of antibodies did not affect the body weight gain of A431 tumor xenografted mice (Fig S1C). The tumor size was measured with calipers by surface approach. Tumor size in the vehicle group exhibited a linear increase throughout the 22-day period and reached size about 1200 mm^3^ (Fig 4B). The injection of cetuximab or MI061 suppressed the tumor growth to 300 mm^3^. Although MI061 was injected more times than other antibodies, it showed significant anti-tumor effect as cetuximab. Each tumor mass raised from xenograft was extracted from the mice to measure relative tumor volume (Fig 4C). Interestingly, injection of MI061 into xenografted mice remarkably suppressed the tumor volume to 25% compared with vehicle-treated group (Fig 4D). Thus, MI061 showed similar anti-tumor effect compared with cetuximab-treat group *in vivo*. Overall, these data supported that purified MI061 from bacterial system can be used for clinical application as an anti-tumor agent.

### The effect of engineered minibody on EGFR downstream signal pathway

**Figure 5:**
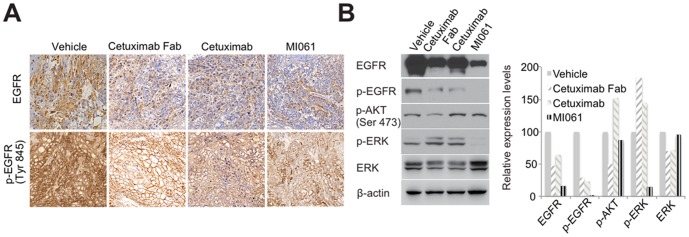
The effect of engineered minibody on EGFR downstream signaling pathway. (A) Phosphorylation of EGFR. The phosphorylated EGFR of frozen section of A431 tumor tissue was analyzed by immunohistochemistry staining using p-EGFR (Tyr 845) antibody. Scale bar = 200 µm (B) EFGR downstream signaling pathway in tumor lysate was analyzed by western blot using each antibody as indicated. β-actin was used as a loading control.

The expression levels of EGFR and phosphorylated-EGFR (p-EGFR) were analyzed from A431 tumor sections as shown in Fig 5A. In comparison with vehicle group, the staining intensities of both EGFR and p-EGFR were remarkably decreased in the all groups treated antibody. To obtain the MI061 effect on EGFR downstream signal pathway, each tumor masses were lysed. They were analyzed for activation status of downstream signaling molecules followed by EGFR activation. Similar to immunohistochemistry data against EGFR and p-EGFR, we found that the expression level of EGFR was significantly reduced in the group treated either CT Fab or MI061 (Fig 5B). In addition, the levels of both p-EGFR and p-ERK were only completely suppressed in the MI061-treated group but not in the other group (Fig 5B). In contrast, CT Fab only inhibited phophorylation of AKT (Fig 5B). These data suggested that CT Fab and MI061 might be involved in different downstream signal pathway, despite a similar inhibitory effect on phosphorylation of EGFR *in vivo*. Taken together, these results demonstrated that higher anti-tumor effectiveness of MI061 compared to cetuximab in A431 xenografted model, supporting the relevance of further studies for the use of MI061 to deliver anticancer agent *in vivo*.

## Discussion

Tumor-targeted therapies using intact whole IgG have been intensively studied for cancer patients in numerous researches and led to a wave of FDA-approved antibodies [6]–[10]. Hence, remarkable outcomes have been achieved in cancer therapy, but there are still some limitations to using whole IgG to treatment in cancer patients. These include extremely high production costs and pharmacokinetics versus tissue penetration [17], [18]. In order to overcome these disadvantages, researchers have been tried to reduce size of antibody for the effective production in bacterial system and enhance the penetration rate of antibody for efficient therapy [16], [19]. In present study, we also generated three minibodies derived from Cetuximab and examined characteristics and anti-tumor effect of them *in vitro* and *in vivo*.

Many attempts have been undertaken to generate antibodies for human therapeutic use in mammalian cells. However, transfected mammalian cells are not preferred as an expression system due to low expression levels, slow growth and expensive nutrient media [20]. These problems can be addressed by using an *E. coli* expression system to produce large quantities of recombinant protein. Although there are some limitations such as intracellular accumulation, the potential for product degradation and production of endotoxin, it has widely been used for recombinant protein production due to its ability to grow rapidly, at high density on inexpensive substrates and relatively simple handling [20]. Also, the various recombinant techniques have enabled production of antibody fragments with various sizes [11], [16], [19], [21]. Minibody (scFv-C_H_3), one of them, can be expressed in bacterial system and their small size and low molecular weights allow fast and easy tissue penetration [22]. In spite of improved technology and heavy research, however, there are still limitations regarding productivity and thermal stability of minibody [22]. Generally, scFvs show low thermal stability and tend to denature or aggregate under the conditions of clinical uses due to the relatively weak V_H_-V_L_ interaction [22]. To conquest low stability, some researchers focused on length of linker, elongation of residue in linker slightly enhanced stability of scFvs [23]. We also tested thermal stability of both MI045 and MI053 and found that MI053, which has 18 residues as a linker, is more stable rather than MI045 during 48 hrs (Fig. S1D and Materials and Methods S1). Some studies have reported that domain orientation of V_L_-V_H_ region on scFvs is structurally important to higher recovery rate of soluble protein [24]. In this work, domain orientation of V_L_-V_H_ region was evaluated for the solubility of minibodies. Interestingly, we found that V_H_-V_L_ orientation is much better than V_L_-V_H_ format to increase solubility of minibody. The reason for the higher purification yield of V_H_-V_L_ arrangement is not clear, but as increasing the stability of scFv has been reported to lead to higher yields of soluble protein [25], it seems to be that V_H_-V_L_ orientation is more stable than V_L_-V_H_ format. Also, since scFVs have tendency to form oligomers, ultrafast liquid chromatography was performed for measuring molecular size of MI061 (Fig. S1A and Materials and Methods S1). After fractionation, fraction number 8 was subjected into western blot analysis. The predicted molecular weight of mature MI061 was 43 kDa including His-tag. As we supplied data in Fig S1B, however, the molecular weight of MI061 was measured around 90 kDa. This data indicates that MI061 forms a stable homo-dimeric complex in the soluble fraction.

Usually, in the xenograft animals, intact IgG antibodies targeting antigens mostly remain in the blood and no more than 20% of the injected dose usually interacts with the tumor [17]. Long lasting half-life of intact IgG in the serum due to its molecular size caused adverse effect as increasing target to non-target binding ratios [11]. Whereas, scFv-based antibody have shown to short half-life in circulation and low binding affinity to target since it is a partial fragment of intact IgG [22], [26]. Here, we found that the half life of MI061 is 4.3 hrs and it has only 3% binding affinity to EGFR. It is relatively short half-life and low binding affinity to target. In principle, it cannot be excluded that the affinity tag may interfere with protein activity, although the relatively small size and charge of the polyhistidine affinity tag ensure that protein activity is rarely affected [27]. Nevertheless, purification of proteins with His-tag is the most commonly used method. And reduced plasma half-life might be advantageous to less exposure of MI061 and to improve target to non-target binding ratios. On the other hand, its low binding affinity is required to increase the dose or frequency of administration and it enables antibody to increased localization and homogenous distribution into solid tumors. Particularly, MI061 showed relatively high affinity compared with other minibodies derived same parental antibody. In addition, tissue penetration rate of antibody is a crucial parameter and it is a rate-limiting step for overall efficiency of the cancer treatment. Typically, the minibody has high tumor specificity and more intensive penetration rate in comparison with intact IgG [28]. Likewise, in this study, MI061 not only showed high tumor specificity in both EGFR competition assay and human protein microarray, but also reduced size of tumor and inhibited phosphorylation of EGFR like as cetuximab.

So far, there is no report describing the antigen binding specificity of engineered minibody and its parental antibody. Both MI061 and cetuximab only interact to EGFR with similar binding capacity but not to other proteins. Generally, cetuximab interacts exclusively with domain III of sEGFR resulting in blocking a ligand binding region and sterically preventing the receptor dimerization [29]. Although cetuximab Fab fragment reduced AKT pathway and MI061 reduced mainly ERK pathway, but they showed a similar inhibitory effect on phosphorylation of EGFR *in vivo*. Since they possess same antigen binding region, it seems to be that cetuximab Fab fragment and MI061 bind exclusively to domain III, covering an epitope that partially overlaps with growth factor binding site on that domain. Although, MI061 showed clearly inhibitory effect as cetuximab *in vivo*, it is necessary to further study characterization of EGFR mediated downstream signaling pathway.

Until now, only two antibodies, trastuzumab and cetuximab have been used for the treatment of solid tumors, whereas over 85% of human cancers are solid tumors. Cetuximab, one of them has been widely used for solid tumors including head and neck cancer. Here, we generated MI061 to overcome the limitations of cetuximab and demonstrated that it is readily produced from bacterial system. Moreover, we also confirmed that it has similar antitumor effect compared to cetuximab in A431 xenograft models. These findings provide a key insight into the development of more effective targeted therapies, emphasizing the importance of minibody as a promising antitumor agent in the cancer therapy.

## Supporting Information

Figure S1 **MI061 forms a stable homo-dimeric complex.** (A). Fractionation analysis of MI061 using a Liquid chromatogram. After chromatography on BioSep-SEC-s2000 column, each fraction was separated on 12% SDS-PAGE and visualized by immunoblot assay with an anti-C_H_3 domain antibody. (B) The molecular weight of MI061 was measured based on the following protein standards: myoglobin (17 kDa), ovalbumin (44 kDa) and IgG (150 kDa). (C) Change in body weights of A431 xenografted mice intraperitoneally injected with antibodies during experiment days. The body weights of mice were measured once every five days. (D) Thermal stability depending on time of engineered minibodies. The minibodies were incubated during 1, 2, 4, 6, 12, 24, 48 hrs at 37°C. The incubated proteins were resolved on 12% SDS-PAGE and visualized by immunoblot assay with an anti-C_H_3 domain antibody.
(TIF)

Materials and Methods S1 **Supplementary Materials and Methods.**
(DOCX)
