# Correction: Effective Therapeutic Approach for Head and Neck Cancer by an Engineered Minibody Targeting the EGFR Receptor

**DOI:** 10.1371/journal.pone.0117895

**Published:** 2015-01-30

**Authors:** The PLOS ONE Staff

The fourth author’s name is spelled incorrectly. The correct name is: Won-Jae Chi. The correct citation is: Kim YP, Park D, Kim JJ, Chi W-J, Lee SH, et al. (2014) Effective Therapeutic Approach for Head and Neck Cancer by an Engineered Minibody Targeting the EGFR Receptor. PLoS ONE 9(12): e113442. doi:10.1371/journal.pone.0113442
