# Supplementary figures and images for: Effective Therapeutic Approach for Head and Neck Cancer by an Engineered Minibody Targeting the EGFR Receptor
Source: PLoS One. 2014 Dec 1;9(12):e113442. doi: 10.1371/journal.pone.0113442 (PMC4249956; doi:10.1371/journal.pone.0113442)

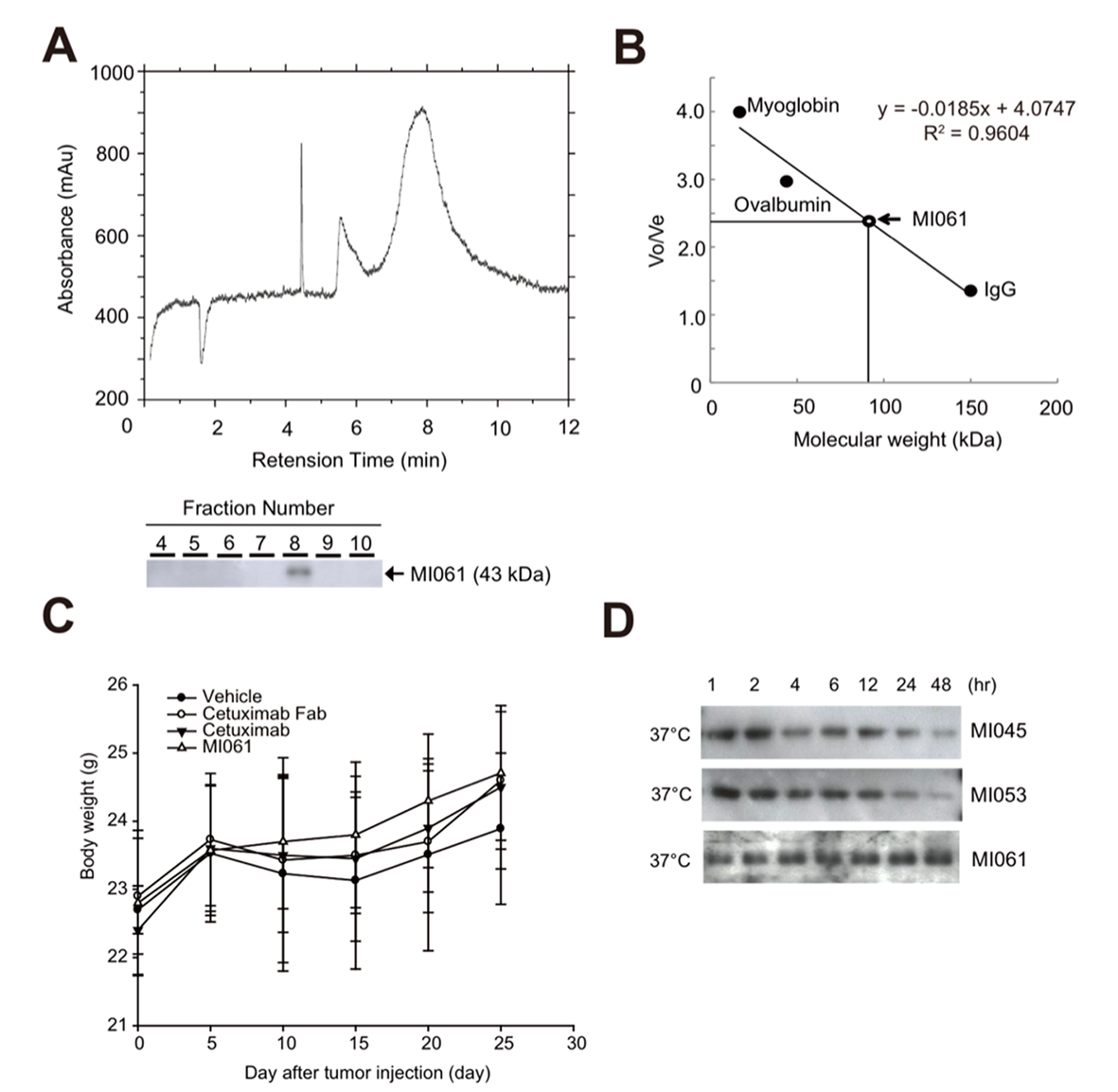

Supplement: Figure S1 — MI061 forms a stable homo-dimeric complex. (A). Fractionation analysis of MI061 using a Liquid chromatogram. After chromatography on BioSep-SEC-s2000 column, each fraction was separated on 12% SDS-PAGE and visualized by immunoblot assay with an anti-CH3 domain antibody. (B) The molecular weight of MI061 was measured based on the following protein standards: myoglobin (17 kDa), ovalbumin (44 kDa) and IgG (150 kDa). (C) Change in body weights of A431 xenografted mice intraperitoneally injected with antibodies during experiment days. The body weights of mice were measured once every five days. (D) Thermal stability depending on time of engineered minibodies. The minibodies were incubated during 1, 2, 4, 6, 12, 24, 48 hrs at 37°C. The incubated proteins were resolved on 12% SDS-PAGE and visualized by immunoblot assay with an anti-CH3 domain antibody. (TIF) [file pone.0113442.s001.tif]
