# Supplementary material for: Effective Therapeutic Approach for Head and Neck Cancer by an Engineered Minibody Targeting the EGFR Receptor
Source: PLoS One. 2014 Dec 1;9(12):e113442. doi: 10.1371/journal.pone.0113442 (PMC4249956; doi:10.1371/journal.pone.0113442)
Supplement: Materials and Methods S1 — Supplementary Materials and Methods. (DOCX) [file pone.0113442.s002.docx]

**Supporting Information**

**Supplementary Materials and Methods**

**Chromatographic separation**

The liquid chromatography system employed was an ultrafast liquid chromatography (UFLC) system (Shimadzu, Kyoto, Japan) equipped with a tunable UV detector (set at 214 nm) for the protein seperations. The column was a 300 mm × 7.8 mm (5 μm) BioSep-SEC-s2000 column (Phenomenex, CA, USA). The mobile phase was 45% acetonitrile with 0.1% trifluoroacetic acid. Flow rate for separations was 1 mL/min. The column was calibrated with the following protein standards: myoglobin (17 kDa), ovalbumin (44 kDa) and IgG (150 kDa). Western blot analysis was conducted on isolated UFLC fractions as described. The isolated samples (12 μl) was initially separated on 12% SDS-PAGE gels and transferred onto a polyvinylidene difluoride membrane (Thermo scientific, USA). The membrane was immunoblotted with primary anti-C_H_3 domain antibody A567H (Thermo Scientific, USA) and each protein was visualized by incubation with secondary HRP-conjugated anti-mouse IgG antibody. The membrane was developed and visualized using Pierce ECL Plus Western Blotting Substrate (Thermo scientific, USA).

**Thermal stability**

To determine the relative thermal stability of three engineered minibodies, they were incubated at 37°C during 1, 2, 4, 6, 12, 24 and 48 hrs. And then, each incubated minibodies were initially separated on 12% SDS-PAGE gels and transferred onto a polyvinylidene difluoride membrane (Thermo scientific, USA). The membrane was immunoblotted with primary anti-C_H_3 domain antibody A567H (Thermo Scientific, USA) and each protein was visualized by incubation with secondary HRP-conjugated anti-mouse IgG antibody. The membrane was developed and visualized using Pierce ECL Plus Western Blotting Substrate (Thermo scientific, USA)
